# Visual snow syndrome and migraine: a review

**DOI:** 10.1038/s41433-023-02435-w

**Published:** 2023-02-14

**Authors:** Elisa Martins Silva, Francesca Puledda

**Affiliations:** 1 Department of Neurology, Hospital Garcia de Orta, Almada, Portugal; 2 Headache Group, Wolfson CARD, Institute of Psychiatry, Psychology and Neuroscience, King’s College, London, UK

**Keywords:** Visual system, Neurological disorders

## Abstract

Visual snow syndrome is a neurological condition characterized by ongoing prominent phenomena described consistently as tiny dots moving across the entire visual field, often associated with complex visual symptoms. These can take the form of afterimages, entoptic phenomena, nyctalopia and light sensitivity. Although some of these symptoms can be benign, they can nonetheless become significantly impactful for many who experience them, particularly in cases that have a sudden and abrupt start. As visual snow syndrome becomes increasingly recognized in clinical practice we begin to learn about its typical presentation and underlying pathophysiology. Treatment of visual snow, however, still proves quite challenging, and efforts need to be focused on unravelling the biological mechanisms of the syndrome. This endeavour has characterized the most recent research on visual snow, mostly involving neuroimaging, neurophysiological and neurobehavioral studies aimed at understanding its underlying neural signature. Another important aspect of the syndrome, which will likely prove critical in deepening our understanding of visual snow, is represented by the intricate biological and historical connexion with migraine. This narrative review focused on visual snow syndrome will explore its clinical, pathophysiological and treatment aspects in detail.

## Abstract (zh)

视雪综合征是一种神经系统疾病, 特征为持续性的在整个视野中移动的小点, 通常与复杂的视觉症状有关。这些症状可能以视觉残像、内视现象、夜盲症和光敏感的形式表现。尽管部分症状可能是良性的, 但它们仍然可能会对经历过这些症状的人造成重大影响, 尤其是在突然发作的情况下。 随着视雪综合征在临床实践中越来越多被认识和鉴别, 我们开始了解其典型的表现和潜在的病理生理学。然而, 视雪综合征的治疗仍然具有相当大的挑战性, 需要聚焦于阐明该综合征的生物学机制。这也是目前视雪综合征研究的主要方向, 主要涉及神经影像学、神经生理学和神经行为学研究, 旨在了解其潜在的神经学特征。 该综合征的另一重要方面是其与偏头痛之间复杂的生物和历史联系, 这可能在加深我们对视雪综合征的理解上至关重要。 这篇关于视雪综合征的综述将详细探讨其临床、病理生理和治疗方面的问题。

## What is visual snow?

Visual snow syndrome (VSS) is a neurological entity characterized by a core symptom, visual snow [1], as well as other bothersome positive visual phenomena. The ‘snow’ is typically described as flickering and moving dots, similar to the static of analogue televisions, that are persistent and occupy the entire visual field [2]. The snow or static can be black and white, transparent, flashing, coloured, or a combination of all of these. The criteria for the full visual snow syndrome were first defined by Schankin and colleagues in 2014 [1] and are now provided in the third edition of the International Classification of Headache Disorders (ICHD-3) [3]. VSS can range in severity and when at its worse, such as when all additional symptoms are present, it can be an extremely disabling chronic disease [4]. On the other hand, some individuals have very mild and non-bothersome symptoms, not requiring medical attention.

In a web-based epidemiological study performed in the UK, VSS prevalence in the general population was found to be around 2% [5], higher than most neurological disorders [6]. The disorder usually starts early in life, and there seems to be no difference in prevalence across genders [7], although some studies have found a slight female predominance (female-to–male ratio of 1.6:1). Increased prevalence in higher age brackets (mean around fifty years) was found in some studies [5] but not others [1, 7, 8]. The clinical presentation of VSS is considered to be similar between different geographic regions, as shown by studies comparing different population of VSS sufferers (such as from the UK and Italy) with very similar sets of symptoms [9]. Prospective studies have shown that VSS symptoms tend to remain stable over time and are not expected to cease [10, 11]. The visual snow itself can, however, manifest episodically, and either remain as an ictal disorder or later evolve into the classic form of VSS [12–14]. In these cases visual snow should carefully be distinguished from visual aura or other visual phenomena associated with migraine [15]. In a large retrospective, single-centre, case series of two hundred and forty-eight cases of visual snow, 15% of patients reported transient symptoms as part of their typical migraine aura [12].

Additional symptoms of VSS include palinopsia, enhanced entoptic phenomena, photophobia (hypersensitivity to light) and nyctalopia (poorer vision in dim light); at least two of these should be present according to the current criteria [1]. Palinopsia in VSS can be described as illusory, meaning that it is caused by the misinterpretation of a real object [16]. It is reported in around 60–80% of patients, either as afterimages, light streaking or trailing of moving objects [7]. Entoptic phenomena occur as observations of physiologic processes from the optic apparatus itself and that are present in great magnitude in VSS patients. They are classified in the syndrome as floaters, blue field entoptic phenomenon, photopsia and self-light of the eye. Excessive floaters in both eyes are, in fact, the most frequently reported additional symptom of VSS [7]. Common entoptic phenomena in visual snow include symptoms such as ‘straight lines moving across the visual field’, ‘water running down a window’ and ‘kaleidoscopes of colours present with eyes open’ [1] even though these are currently not part of the definition of VSS [17].

The majority of cases of VSS are represented by benign primary VSS. There are, however, several secondary causes that can present as mimics of visual snow and which should therefore be considered in the differential diagnosis. Mehta and colleagues describe that an inciting event or comorbid illness can be present in 40% of patients with visual snow [12]; this is particularly relevant since secondary forms may be characterized by different prognoses and treatment options with respect to primary visual snow. In fact, the visual snow phenomenon can underlie serious ophthalmological [18] or brain disorders [19, 20], which should be thoroughly investigated and excluded, at a minimum with standard history, ophthalmologic examination, visual fields testing and optical coherence tomography (OCT).

Although patients with primary idiopathic VS and VSS will have unremarkable ophthalmology [21, 22], presentations due to underlying ophthalmologic causes have been described, and therefore a careful ophthalmic examination of the optic apparatus and pathways is always required. Such cases range from retinitis pigmentosa [23], to rod-cone dystrophy [21], autoimmune/paraneoplasic retinopathy such as glycine receptor antibody syndrome [24], uveitis, macular atrophy, central serous retinopathy and vitreous detachment [12]. In certain cases where less common causes are suspected, particularly with sudden symptom onset, then further testing for conditions such as bilateral optic neuropathies, methanol intoxication, ischemia, Leber Hereditary Optic Neuropathy, and vitamin deficiencies should be pursued, Neurological causes such as idiopathic intracranial hypertension, prion disease and other structural lesions of the posterior visual pathway should also be considered [12, 15].

In general, when VS or VSS presents non-progressively at an early age, with typical symptoms and normal neurologic and ophthalmologic (including automated perimetry and macular OCT) examinations, further workup is likely not necessary. Other investigations, including electroretinogram and magnetic resonance imaging (MRI), should be considered in new onset cases, if the symptoms are not completely fitting the diagnostic criteria or if the patient’s history changes over time [25].

Another disorder to consider in the differential diagnosis of VSS is Hallucinogen Persisting Perception Disorder (HPPD) [7]. This is a complex condition which can occur after the intake of different recreational drugs, such as lysergic acid diethylamide, ecstasy and psylocibin [26] and that is characterized by intense ‘flashbacks’, particularly of visual nature [27]. These can be very similar to the presentation of primary VSS, although HPPD presenting with visual snow appears to be more common in males and has a later onset of symptoms [7]. Interestingly, migraine is not frequently reported in these patients, suggesting that different pathophysiological mechanisms with respect to primary forms are at play [28]. Nonetheless, both HPPD and VSS lack good therapeutic approaches, making management of these patients highly challenging [29].

The main comorbidities of visual snow are migraine and tinnitus. Bilateral, continuous, and non-pulsatile tinnitus is reported in up to 75% of visual snow patients [7], making it almost a hallmark feature of the syndrome. It is hypothesized that visual snow and tinnitus are intertwined manifestations of a similar perceptual anomaly. This abnormality, of which the origin is largely unknown, could be leading the brain to perceive phantom sensations even when outside stimuli are absent or subthreshold.

There is currently a lack of evidence for effective treatment of VSS, therefore management includes reassurance on its benign nature and dealing with associated comorbidities. In this sense, particular attention needs to be placed on psychiatric conditions, as these can be very distressing and have shown to lower patients’ quality of life [30].

Lamotrigine is commonly tried in clinical practice, based on the anecdotal evidence of complete remission of symptoms following its use, in one case report [31]. This drug also has the added benefit of improving depersonalization symptoms, which are frequent in VSS [30, 32]. Notwithstanding, data on the pharmacology of visual snow from larger datasets remains disappointing [8, 10] with lamotrigine itself showing efficacy in only 20% of patients, with no reported remission. A perspective web-based study showed that most patients report no effect with any medication and can even have worsening of symptoms with lamotrigine (35%) or other commonly used drugs. Interestingly, this study showed an occasional benefit with vitamins and nutraceuticals, which can therefore be considered in some patients given their high safety. The category of benzodiazepines and hypnotics also led to a small net benefit, warranting further investigation given that these medications do come with a low tolerability profile [10]. Finally, this study provided evidence on substances that VSS patients should avoid whenever possible, as they can have a deleterious effect, including recreational drugs, alcohol, atypical antidepressants and ADHD medication.

Non-pharmacological interventions for VSS include filtered lenses, particularly within the blue-yellow colour spectrum [33] and repetitive transcranial magnetic stimulation [34], however, evidence on these treatments is still lacking.

## Migraine and VSS

Since its first description, visual snow has been entangled with the disease of migraine [35]. VSS was first regarded as a rare complication of this much more prevalent disorder, and only later recognized as a clearly distinct condition [1]. Importantly, even if the two are often comorbid, with migraine biology aggravating the clinical phenotype of VSS [36], routinely used treatments for migraine offer little to no benefit in VSS [1].

Photophobia, the perception of normal light being either too bright or painful, is a hallmark symptoms of both migraine and VSS, representing an important pathophysiological and clinical link [37]. Light sensitivity can be present in migraine both during attacks and interictally [38], and a continuous hypersensitivity to light is reported in more than half of VSS individuals [1, 7]. In fact, in a case-control study, VSS photophobia was shown to be independent of concomitant migraine, with levels of perceived visual sensitivity similar to those of chronic migraine patients [39].

Migraine photophobia seems to be mediated by alterations in the retino-thalamo-cortical pathway [40] and has also been linked to visual cortex hyperexcitability [41]. In a study investigating migrainous photophobia using H_(2)_^(15)^O PET, it was revealed that an area of the extrastriate visual cortex mostly involving the lingual gyrus (LG) shows increased activation during light stimulation in patients [42]. This same brain area, involved in relevant physiological functions such as visual memory, has found to be abnormally active in VSS as well [36]. In fact, a ^18^F-FDG PET investigation showed hypermetabolism of the lingual gyrus in subjects with VSS when compared to healthy controls [43]. In a more recent study, response to a visual stimulation mimicking visual snow was used to study underlying pathophysiological changed of the VSS brain [44]. Using combined functional neuroimaging and magnetic resonance spectroscopy, a disturbance in anaerobic metabolism in the right lingual gyrus was found in patients, thus confirming the involvement of this area in the underlying biology of VSS. The study also showed a negative correlation between lactate concentrations and BOLD responses after visual stimulation, suggesting an overall disturbance in the downstream processing of visual inputs in VSS, which might explain photophobia.

Another important aspect of the relationship between migraine and visual snow is represented by aura. Visual symptoms are the most common presentation of aura and include positive or negative visual phenomena, as well as visual perception disturbances. Aura has been found to occur more frequently in subjects with VSS [5, 7] with respect to the general population [45], however, unlike with migraine, the presence of aura alone is not necessarily linked to a more severe phenotype of visual snow [36]. Indeed, if aura is less likely to cause worse manifestations of VSS, then perhaps the increased association between the two conditions is a truly biological one, rather than a ‘simple’ co-occurrence in which each condition causes the other to be more severe.

## VSS pathophysiology and conceptual framework

In the last few years, different aspects of visual snow syndrome pathophysiology have been investigated, mostly through the use of complex techniques involving the fields of neuroimaging, neurophysiology, brain behaviour and neuro-ophthalmology.

### Neuroimaging

With regards to neuroimaging, other than the already mentioned hypermetabolism of the lingual gyrus [43, 44], structural studies using voxel-based morphometry have demonstrated increased grey matter volume in this cortical area [43, 46]. Further, one case series showed hypoperfusion in the temporo-occipital area corresponding to the ventral visual stream (and thus also including the LG) in one patient with VSS [47].

Functional MRI studies have further shown significant alterations in the functional connectivity of different brain systems in VSS, including the visual system, the pre-cortical and cortical visual pathways, the visual motion network, the attentional and salience networks [48]. Involvement of the salience network, which has a role in selecting relevant sensory stimuli for higher-order brain areas, has also been demonstrated by reduced anterior insulae activity to an external visual stimulus in VSS [44].

Increased cortical activation in VSS, not limited to the visual cortex, has been exemplified through the use of arterial spin labelled MRI [49]. Cortical areas of increased cerebral blood flow—an indirect measure of neuronal activation—seem to include the bilateral striate and extrastriate visual cortices, motor cortices, the posterior cingulate cortex and the cerebellum. These findings also seem to occur regardless of the brain state of patients, either at rest or when subject to an active visual stimulus.

Using voxel-based morphometry and guided by PET changes, a recent study showed increased grey matter volume in the extrastriate cortex, temporal and limbic lobes, as well as decreased volume in the superior temporal gyrus in patients with VSS [43]. The latter finding was speculated to be related to concomitant tinnitus, which is an important consideration to make when studying the condition through neuroimaging. Another study also showed grey matter volume changes in areas both internal to the visual system—V1, visual motion area V5—and involving lobule VI/Crus I of the cerebellum in patients with VSS matched to healthy controls [50]. Furthermore, widespread white matter abnormalities have been reported in VSS [51], dispersed within and outside the visual cortex, in areas involved in visual processing and visual conceptualization [52].

### Neurophysiology

Electrophysiological studies have attempted to uncover patterns of neuronal activation in VSS. Looking at occipital cortex hyperexcitability with transcranial magnetic stimulation, Yildiz and colleagues found an increased phosphene threshold in VSS patients, as well as decreased visual habituation, which authors hypothesized to represent a marker of neuronal hyperexcitability of the visual pathway [53]. A study using TMS to excitation/inhibition within the primary visual cortex, however, found no particular changes in VSS with respect to patients with migraine [54]. The same authors, using visual evoked potentials, found that patients with VSS presented an increased N145 latency compared to controls and migraineurs, possibly suggesting abnormal processing in the extrastriate visual cortex [55]. On the other hand, this group could not find elements to suggest altered habituation in patients with VSS.

More research is needed in the future to better understand the mechanisms of neurophysiology that characterize VSS, and to elucidate these inconsistencies across studies.

### Neuro-ophthalmology

Several types of visual tasks have been used to study changes in oculomotor behaviour and visual processing in VSS.

One study was able to uncover a deficit in spatial contrast sensitivity measured through visual perception thresholds [21], in patients with VSS. McKendrick and colleagues applied four different visual tasks targeting visual processing in visual snow and showed an imbalance between excitation and inhibition in primary visual areas [56].

These results were confirmed by the same group when comparing visual contrast perception in patients with VSS with and without migraine, healthy controls and migraineurs, finding what can be described as increased neural responses to external stimuli in VSS [57].

The integrity of the ocular motor network has also been investigated in VSS, with one study revealing faster eye movements in oculomotor task towards novel stimuli, regardless of the difficulty of the test [55], thus suggesting poor attentional control in VSS. This was hypothesized by the authors to be caused by abnormal communication within thalamo-cortical processing networks [56], in further work. VSS patients also present specific saccadic behavioural profiles that are unique to the condition, suggesting that these tests might represent reliable instruments to evaluate the disorder in an objective manner [58].

Overall, findings from these studies collectively show a widespread processing disorder in VSS, which involves different brain networks and is not limited to the visual cortex [59]. Further, the clinical and epidemiological overlap with migraine, aura and tinnitus, suggest that these changes are common to different disorders, that being connected to visual snow possibly represent a spectrum of altered disturbances of sensory processing.

## Conclusions

Visual snow is a phenomenon of central nervous system origin characterized by the perception of a continuous visual static. The visual snow syndrome is a more complex entity in which the static is accompanied by prominent visual symptoms, that often co-occurs with migraine, aura, and tinnitus.

The underlying mechanisms causing visual snow syndrome, although not fully understood, appear to be linked with dysfunctional sensory processing and altered occipital cortex excitability. As pharmacological treatment for visual snow syndrome is mostly ineffective, patient management should be focused on reassurance, appropriate differential diagnosis, search of possible secondary causes in atypical cases and optimal handling of associated comorbidities.
